# The impact of *O*-glycan chemistry on the stability of intrinsically disordered proteins†
†Electronic supplementary information (ESI) available: Methods for synthesis and thermolysin digestion of linker variants, details of the simulations and analyses, and additional results. See DOI: 10.1039/c7sc05016j


**DOI:** 10.1039/c7sc05016j

**Published:** 2018-03-20

**Authors:** Erica T. Prates, Xiaoyang Guan, Yaohao Li, Xinfeng Wang, Patrick K. Chaffey, Munir S. Skaf, Michael F. Crowley, Zhongping Tan, Gregg T. Beckham

**Affiliations:** a National Bioenergy Center , National Renewable Energy Laboratory , Golden , CO 80403 , USA . Email: gregg.beckham@nrel.gov; b Institute of Chemistry , Center for Computational Engineering and Sciences , University of Campinas , 13084-862 , SP , Brazil; c Department of Chemistry and Biochemistry and BioFrontiers Institute , University of Colorado , Boulder , CO 80303 , USA . Email: zhongping.tan@colorado.edu; d Biosciences Center , National Renewable Energy Laboratory , Golden , CO 80403 , USA . Email: michael.crowley@nrel.gov

## Abstract

Protein glycosylation is a diverse post-translational modification that serves myriad biological functions. *O*-linked glycans in particular vary widely in extent and chemistry in eukaryotes, with secreted proteins from fungi and yeast commonly exhibiting *O*-mannosylation in intrinsically disordered regions of proteins, likely for proteolysis protection, among other functions. However, it is not well understood why mannose is often the preferred glycan, and more generally, if the neighboring protein sequence and glycan have coevolved to protect against proteolysis in glycosylated intrinsically disordered proteins (IDPs). Here, we synthesized variants of a model IDP, specifically a natively *O*-mannosylated linker from a fungal enzyme, with α-*O*-linked mannose, glucose, and galactose moieties, along with a non-glycosylated linker. Upon exposure to thermolysin, *O*-mannosylation, by far, provides the highest extent of proteolysis protection. To explain this observation, extensive molecular dynamics simulations were conducted, revealing that the axial configuration of the C2-hydroxyl group (2-OH) of α-mannose adjacent to the glycan–peptide bond strongly influences the conformational features of the linker. Specifically, α-mannose restricts the torsions of the IDP main chain more than other glycans whose equatorial 2-OH groups exhibit interactions that favor perpendicular glycan–protein backbone orientation. We suggest that IDP stiffening due to *O*-mannosylation impairs protease action, with contributions from protein–glycan interactions, protein flexibility, and protein stability. Our results further imply that resistance to proteolysis is an important driving force for evolutionary selection of α-mannose in eukaryotic IDPs, and more broadly, that glycan motifs for proteolysis protection likely coevolve with the protein sequence to which they attach.

## Abstract 2

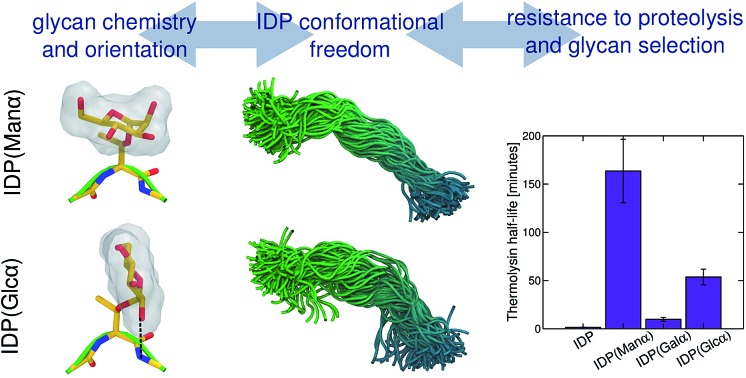
Protein glycosylation is a diverse post-translational modification that serves myriad biological functions.

## 

Intrinsically disordered proteins (IDPs) and intrinsically disordered regions (IDRs) of proteins are prevalent in both eukaryotes and prokaryotes.^1^–^3^ Although often poorly conserved in sequence, the amino acid content of IDPs and IDRs is actively regulated, and IDPs and IDRs serve functions such as connecting ordered domains, regulating translation, molecular recognition and signaling, and assisting in protein folding.^2^–^4^ Because of their inherent flexibility and lack of structure, IDPs and IDRs are susceptible to proteolytic cleavage in the competitive, extracellular milieu, and *O*-glycosylation – the attachment of a sugar moiety to the β-hydroxyl group of serine or threonine – is an important mechanism to protect against proteolysis in these regions.^5^ In fungi and yeasts in particular, most of the secreted IDPs and proteins exhibiting IDRs are *O*-mannosylated,^6^–^9^ but the evolutionary preference for this specific glycosylation pattern is not well understood. The present study uses glycopeptide synthesis and molecular dynamics (MD) simulations to reveal that *O*-mannosylation is the preferred glycan motif on fungal IDP sequences and reveals the biophysical reasons underpinning this observation, in turn suggesting an evolutionary selection for α-mannose as the preferred glycan for IDP/IDR stabilization in some eukaryotic systems.

*O*-Mannosylation is strongly preferred for proteolysis protection of a model fungal IDP. To investigate how glycan identity affects IDP proteolytic stability, we employed the naturally *O*-mannosylated linker from the *Trichoderma reesei* glycoside hydrolase family 7 cellobiohydrolase, *Tr*Cel7A, as a model.^10^ This enzyme is one of the most important industrial cellulases and its linker is a well-studied *O*-mannosylated IDP.^11^–^14^ The α-anomeric configuration was chosen since it is the only type reported so far in reducing terminal mannose residues of *O*-mannosylated proteins from fungi and yeasts.^8^ We used solid-state glycopeptide synthesis^12^,^15^,^16^ to produce four variants (Fig. 1), including the non-glycosylated linker, and measured the half-life to thermolysin degradation with MALDI-TOF MS (Fig. S1–S4†).^15^–^19^ As shown in Table 1, all glycosylated variants improve proteolytic stability over the non-glycosylated linker, L_NG_, but the *O*-mannosylated linker (L_man_) exhibits an striking 112-fold improvement over L_NG_, 16-fold proteolysis protection over the *O*-galactosylated linker (L_gal_), and 3-fold over the *O*-glucosylated linker (L_glc_). These results, obtained using a model IDP, align with our previous observation that *O*-mannosylation improves proteolytic stability compared to other glycans in an ordered protein domain from the same enzyme.^15^,^16^

**Fig. 1:**
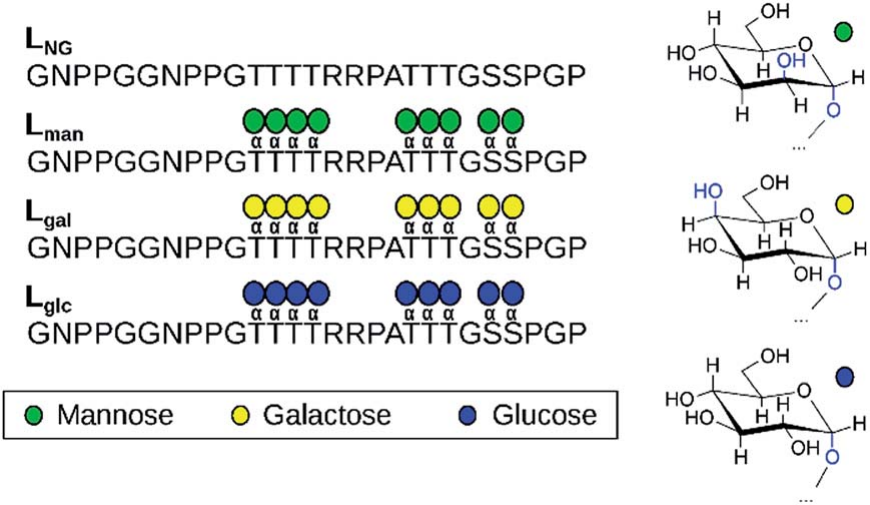
The four linker models examined experimentally and computationally (left). Chair representations of α-mannose, α-galactose, and α-glucose are also depicted (right).

**Table 1:** Half-life to thermolysin degradation (minutes)

Variants	Trial 1	Trial 2	Trial 3	Average
L_NG_	1.6	1.5	1.2	1.5 ± 0.2
L_man_	163.9	196.4	130.5	163.6 ± 32.9
L_gal_	8.5	8.8	12.1	9.8 ± 2.0
L_glc_	53.3	62.0	45.9	53.7 ± 8.1

Glycan stereochemistry impacts protein flexibility and accessibility. To explain the results presented in Table 1, we subsequently conducted temperature replica exchange molecular dynamics (T-REMD) with explicit solvent using various linker models, including the four experimental systems.

Analyses are reported on the T-REMD population from the lowest temperature replica (300 K). Two hypotheses for the increased proteolytic stability imparted by glycans are that (i) glycans increase protein rigidity^20^,^21^ and that (ii) glycans impart steric hindrance to restrict protease access.^22^ Both hypotheses were tested computationally by examining differences in protein flexibility and accessibility. Notably, the predicted cleavage sites to various proteases coincide with the glycosylation sites (Fig. S5†), perhaps suggesting that steric hindrance may be responsible for proteolysis resistance. However, the calculated solvent accessible surface area is similar for all glycosylated models considered (Fig. S6†), while there is a considerable difference in proteolysis susceptibility among L_man_, L_gal_, and L_glc_, with L_gal_ exhibiting only slightly higher resistance to proteolysis than L_NG_. These results suggest that steric hindrance alone cannot fully explain proteolytic resistance, since the glycan moieties occupy roughly the same volume.

We subsequently examined how glycan chemistry affects protein flexibility, glycan orientation, specific interactions, and backbone torsional preferences in an attempt to explain the high proteolysis resistance imparted by *O*-mannosylation. Information about protein flexibility and extension were obtained from the free energy profiles, or potential of mean force (PMF), as a function of the end-to-end distance for all linkers (Fig. 2). Unlike L_gal_ and L_glc_, for which the PMFs are somewhat flat-bottomed and resemble that of the non-glycosylated linker L_NG_, the PMF for L_man_ is slightly narrower and shows a well-defined local minimum at larger distances (∼3.0–3.5 nm). This indicates that L_man_ is, on average, stiffer and adopts more extended conformations than its counterparts. Further analyses reinforce the hypothesis that α-mannosylation is able to restrict protein flexibility. That is, the relative stiffening of L_man_ was corroborated by its greater persistence length (Table S1†). Also, similar structures from T-REMD were clustered considering the Cα atoms with a root mean squared deviation cutoff of 1.5 Å (Fig. S7, Table S3†).^23^ The most populated clusters were found for L_man_. Moreover, values of root-mean-square deviation relative to average structures computed for 10 ns trajectory blocks also indicate lower mobility of the L_man_ backbone (Table S3†). Small differences in protein backbone flexibility and concomitant large differences in resistance to proteolysis were also recently found for a structured protein with a single attached glycan, α-mannose or α-glucose.^24^ Chaffey *et al.* suggested that a chain of specific interactions between *O*-mannosyl and side chains of close residues may be propagating stiffening along the protein backbone. The similar behavior observed with IDPs suggests that the effects of α-mannose on protein stiffening may not be exclusive to a specific protein fold. From these observations, we further hypothesized that the observed differences in linker extension are caused by local interactions with the C2-hydroxyl group (2-OH) adjacent to the glycan–peptide bond, which is equatorial in α-glucose and α-galactose and axial in α-mannose. Fig. 3A shows the average number of hydrogen bonds (HBs) between the protein and each of the carbohydrate hydroxyl groups computed from the T-REMD simulations. The HBs between the 2-OH group and the peptide contribute significantly to the higher total number of HBs in L_gal_ and L_glc_. Compared to L_man_, this indicates that the equatorial configuration of 2-OH, the closest hydroxyl to the peptide chain, favors glycan–protein HBs.

**Fig. 2:**
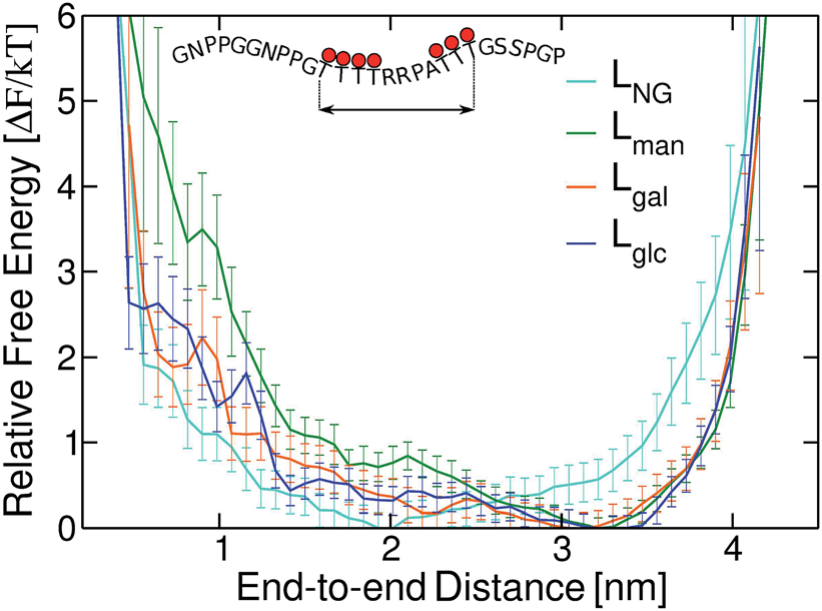
Free energy profiles as a function of the end-to-end distance of *Tr*Cel7A linkers. Error bars were computed with bootstrapping analysis.

**Fig. 3:**
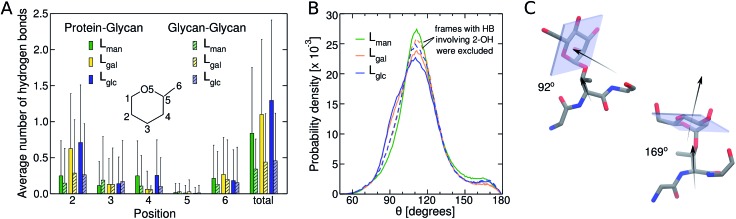
(A) Average number of HBs involving hydroxyl groups in the different positions of the glycan ring. Solid and striped bars correspond to glycan–peptide and glycan–glycan interactions, respectively. Vertical lines indicate standard deviations; (B) probability distribution of the angle between the normal to the plane of the carbohydrate ring and the vector between Cα and Cβ belonging to the threonine to which the glycan is bound. The dashed lines correspond to the distributions resulting from trajectories without the frames with HBs between 2-OH and the protein; (C) representative structures for ∼90° and ∼170° angles obtained for L_man_.

Next, we show that orientation of the glycans relative to the peptide chain depends on the glycan chemistry and affects the conformational freedom of the glycosylated IDP. Fig. 3B shows the normalized distribution of the angle *θ* between the normal to the plane of the sugar ring and the vector formed by Cα and Cβ of the threonine residues to which the glycan is attached. Values near 180° and 90° correspond, respectively, to conformations in which the plane of the rings are nearly parallel and perpendicular to the direction of the peptide chain (Fig. 3C). The shoulder at ∼90° observed for L_gal_ and L_glc_ indicates that the glycans are more frequently oriented perpendicularly to the peptide chain than in L_man_, and, therefore, exhibit smaller contact surface with the protein (Table S2†). This effect is associated to the pronounced glycan–protein HBs involving the equatorial 2-OH in L_gal_ and L_glc_. The normalized angle distributions computed for the subset of molecular frames in which these specific interactions are absent (Fig. 3B, dashed lines) lack the characteristic shoulder in the 80–100° range, demonstrating that the C2 stereochemistry impacts the glycan conformation.

Taken together, the results presented thus far demonstrate that the 2-OH position affects glycan conformation and that protein dynamics differ depending on glycan chemistry. Next, why α-mannosylation leads to more extended conformations and reduces protein flexibility requires an explanation. To this end, we examined how glycans affect the protein backbone conformational sampling at the residue level. Fig. 4A shows the Ramachandran plots for the L_NG_ threonines, in which the protein backbone frequently visits all three major conformational regions. The R3 region corresponds to α-helix like conformations, whereas R1 and R2 correspond to more extended conformations, such as those found in β-sheets and polyproline II structures. Although no persistent secondary structures were detected during the simulations, these results reflect the structural features of the linkers. We verified that attached glycans alter torsional sampling of the nearest amino acids, as seen elsewhere.^25^,^26^ For L_gal_ and L_glc_, the same three regions are populated as in L_NG_, except that the peak in the R3 region occurs only every other residue because of the excluded volume of neighboring glycans (Fig. 4C and D, S8†). In contrast, the R2 region is predominantly favored in L_man_ for all glycosylated residues, suggesting that the relative rigidity of the α-mannosylated linker results in part from a reduced local dihedral flexibility of the glycosylated residues imparted by α-mannosylation (Fig. 4B). We suggest that perpendicularly oriented glycan rings in L_gal_ and L_glc_ allow for improved accommodation of neighboring glycan rings, favoring more compact conformations. Conversely, the preferred orientation of α-mannose glycans hinders the mobility of the surrounding atoms in the peptide chain, thus revealing a direct relationship between glycan chemistry, orientation, and protein conformational freedom.

**Fig. 4:**
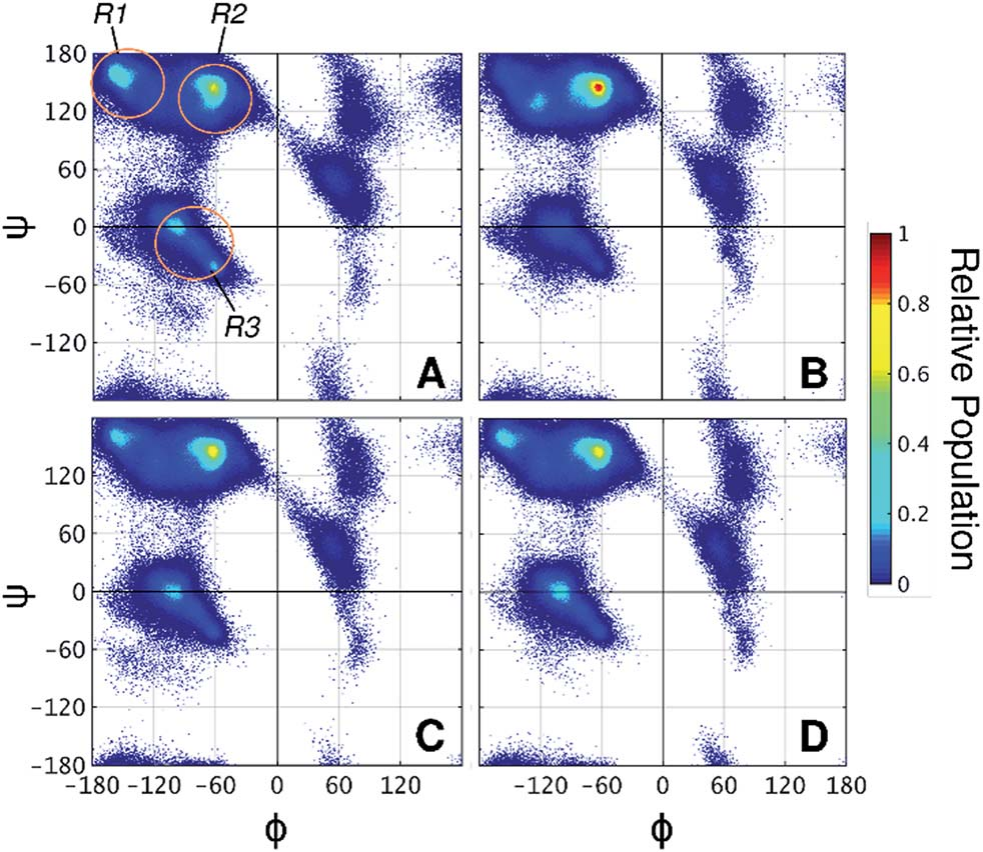
Ramachandran plots of threonine residues in (A) L_NG_, (B) L_man_, (C) L_gal_, and (D) L_glc_. R1, R2, and R3 regions are indicated on panel A. Angles are presented in degrees.

Variants decorated with *O*-mannobiosyl (L_2man_) or *O*-galactobiosyl (L_2gal_) were also simulated, as well as the linker with a putative natural decoration based on a previous experimental characterization (L_man-h_) (Fig. S9 and S10†).^10^ Our analyses suggest that the length of the glycan only slightly changes the dynamics of the protein when the chemistry of the 2-OH groups in the immediately attached glycosyl unit is preserved, reinforcing its importance (Fig. S11†).

Glycosylation pattern and protein primary sequence are correlated. Although less well studied, many secreted bacterial proteins are also *O*-glycosylated.^27^ For example, the multi-enzyme cellulosome from *Clostridium thermocellum* exhibits *O*-glycans on its linkers.^28^ Similarly, the thermostable enzyme CelA from *Caldicellulosiruptor bescii* has linkers of up to 70 amino acids rich in *O*-glycans.^29^ However, unlike the typical *O*-mannosylated linkers from eukaryotic proteins, these linkers exhibit mostly *O*-galactosylation, and are enriched in proline, relative to eukaryotic IDRs.^30^

Aiming to understand why *O*-mannosylation is not prevalent in bacterial IDP and IDRs relative to their eukaryotic counterparts, we also studied a “PT linker”, which comprises a proline–threonine repeat sequence, and represents a fragment of glycosylated linkers found in bacterial cellulases.^20^,^28^,^29^,^31^,^32^ PT linker models were uniformly decorated with α-mannose (L_PT-man_), α-galactose (L_PT-gal_), and α-glucose (L_PT-glc_) (Fig. 5A).

**Fig. 5:**
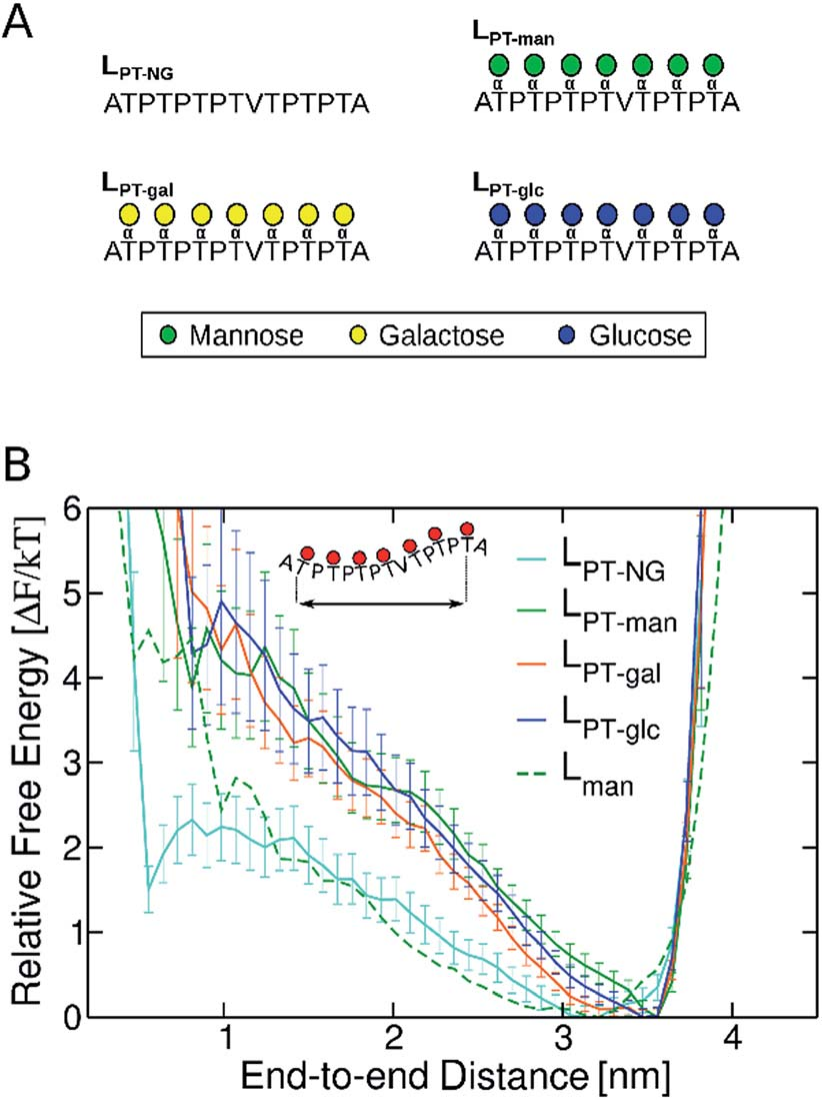
(A) Non-glycosylated (L_PT-NG_) and glycosylated variants of “PT linker” (L_PT-man_, L_PT-gal_ and L_PT-glc_). (B) Free energy profiles as a function of the end-to-end distance of PT linkers. Error bars were computed with bootstrapping analysis. The free energy profile of L_man_ was computed for the distance between Cα atoms in residue 10 (G) and residue 22 (G), so that fragments of same length can be compared.

It is well known that high proline content is generally found in disordered proteins^33^ and favors extended conformations of IDRs.^34^ Accordingly, the end-to-end distance PMF shows that the non-glycosylated PT linker favors extended conformations similarly to the glycosylated *Tr*Cel7A linker L_man_ (Fig. 5B). Elongation and further stiffening of the linkers are observed upon glycosylation and is consistent with NMR spectroscopy data,^34^ which demonstrated that glycosylation of PT linkers dampens the dynamics. Interestingly, in the PT linkers, varying the glycan chemistry is not as impactful to the protein dynamics as in the eukaryotic linker cases. To understand this difference, we examined the correlation between protein dynamics and carbohydrate structuring proposed from the findings with the eukaryotic linker models. In the PT linkers, the presence of the equatorial 2-OH groups in galactosylated and glucosylated linkers does not increase the number of protein–glycan HB compared to L_man_ nor favor perpendicular ring orientations, unlike L_gal_ and L_glc_ (Fig. S12†). Moreover, the Ramachandran plots of threonines are remarkably similar for the three glycosylated PT linkers (Fig. 6), and show the same preference for extended conformations as L_man_ does (R2 region). Together, these results predict that the C2 hydroxyl stereochemistry is unlikely to impact proline-rich IDPs. That may result from the loss of one of the HB sites in the protein backbone, since the backbone nitrogen atom is part of the pyrrolidine ring of proline residues.

**Fig. 6:**
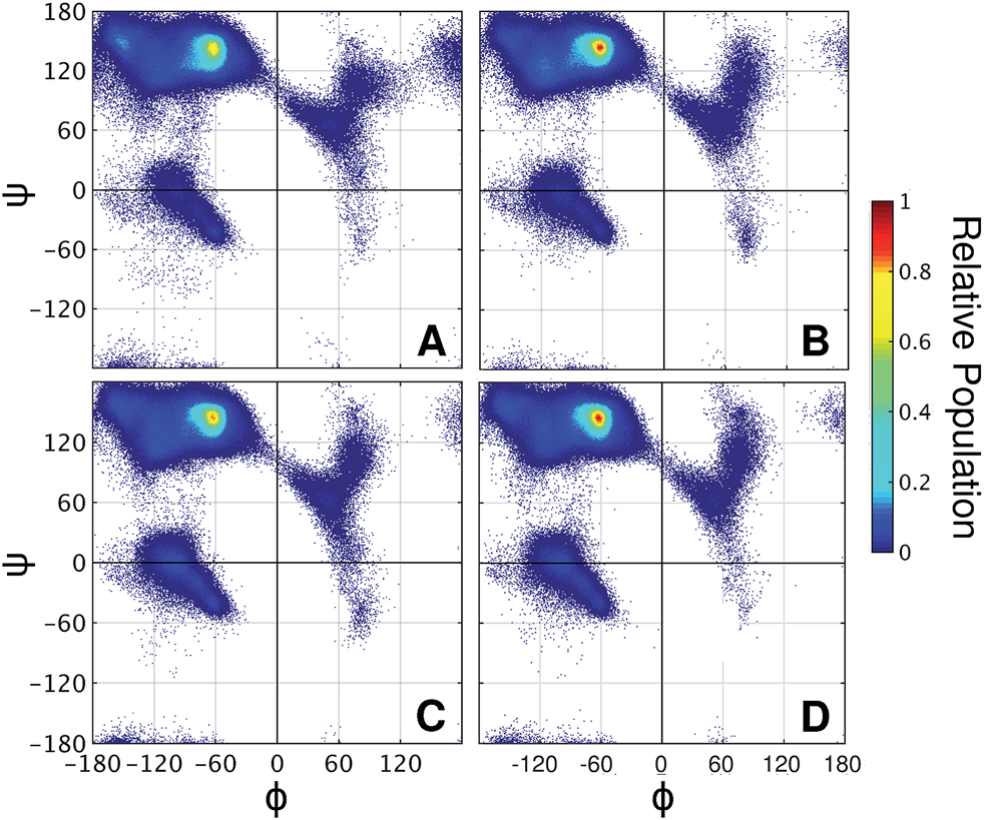
Ramachandran plots of threonine residues in the variants of PT linker (A) L_PT-NG_, (B) L_PT-man_, (C) L_PT-gal_, and (D) L_PT-glc_.

T-REMD simulations of glycosylated tripeptides GTG were also performed to evaluate the effects of 2-OH configuration on glycan orientation and interactions without the influence of neighboring glycans and amino acids. A single glycan, α-mannose, α-galactose or α-glucose, was *O*-linked to the central threonine in the models T_man_, T_gal_ and T_glc_, respectively (Fig. S9†). The parallel glycan-peptide backbone orientation is favored in the small model systems with α-*O*-mannosylation, T_man_, relative to other glycans (Fig. S13†).

In the tripeptides T_gal_ and T_glc_, the equatorial configuration of 2-OH in α-Gal and α-Glc favors HB interactions with the peptide as in the L_gal_ and L_glc_ linkers. However, an excess of perpendicularly-oriented glycans relative to L_man_ is not observed for these tripeptides, indicating that the local HB interactions between 2-OH and the peptide are not the only factor affecting glycan conformation. Instead, these results indicate that the glycans in L_gal_ and L_glc_ are primarily perpendicularly oriented because of the excluded volumes of neighboring glycans and amino acid side chains, and that the 2-OH—peptide HBs stabilize this glycan conformation. Thus, our results with the small tripeptides suggest that the primary sequence and the distribution of glycosylated residues along the peptide chain are important factors for carbohydrate orientation in these systems.

In summary, experimental comparisons of glycosylated and non-glycosylated IDPs show that *O*-mannosylation enhances protection against proteolysis by two orders of magnitude relative to the non-glycosylated parent IDP, followed by *O*-galactosylation (10-fold improved stability). Our results suggest that the resistance to proteolysis is an important driving force for the natural selection of α-mannose as the main *O*-linked glycan motif decorating IDRs and IDPs in secreted eukaryotic proteins. Furthermore, these results demonstrate that the stereochemistry of C2 in the carbohydrate rings plays a key role on glycan orientation, which is correlated to protein flexibility and extension. Accordingly, the axial position of 2-OH in an α-mannose glycan is related to the observed higher rigidity and extension of the studied IDR. While associating protein elongation with resistance to proteolysis is perhaps counterintuitive, protein stiffening can explain the remarkably higher stability of the *O*-mannosylated linker. That is, although we have not investigated the interactions between a protease and IDPs, we conjecture, in the light of the present findings, that increasing the peptide rigidity impairs binding to the catalytic site of a protease. This hypothesis is reinforced by the observation of a similar trend of glycan chemistry impacting resistance to proteolysis of a structured protein and its thermal stability, which is often linked to protein stiffening.^16^ Moreover, the effect of glycosylation on the average elongation of the studied IDR, as a protein linker, may be important to provide the optimum distance between the connected domains for protein function. Therefore, *O*-linked α-mannose exhibits the unique ability of both extending the IDR while protecting it against proteolysis.

These results also suggest that the high content of proline residues, especially found in linkers from bacterial cellulases, avoids the need for α-mannose for increased protection against proteolysis. This hypothesis will be tested in future experimental studies. We further suggest that the glycosylation pattern in eukaryotic IDRs co-evolved with the primary sequence. That is, the lower content of proline residues in IDPs and IDRs from fungi compared to bacteria is compensated by *O*-linked α-mannosylation to guarantee optimal linker length, flexibility, and protection against proteolysis. Given the compelling alignment of experimental and computational results, we anticipate that our findings will be useful in the burgeoning field of glycoprotein engineering.

## Conflicts of interest

There are no conflicts to declare.

## Supplementary Material

Supplementary information
