# Supplementary material for: The impact of O-glycan chemistry on the stability of intrinsically disordered proteins
Source: Chem Sci. 2018 Mar 20;9(15):3710–5. doi: 10.1039/c7sc05016j (PMC5939190; doi:10.1039/c7sc05016j)
Supplement: Supplementary file 1 [file SC-009-C7SC05016J-s001.pdf]

Electronic Supplementary Material (ESI) for Chemical Science.
This journal is © The Royal Society of Chemistry 2018

Supporting Information

The impact of *O*-glycan chemistry on the stability of intrinsically disordered proteins

Erica T. Prates[a,b], Xiaoyang Guan[c], Yaohao Li[c], Xinfeng Wang[c], Patrick K Chaffey[c], Munir S. Skaf[b], Michael F. Crowley[*,d], Zhongping Tan[*,c], Gregg T. Beckham[*,a]

[a] National Bioenergy Center, National Renewable Energy Laboratory, Golden CO 80403 United States

[b] Institute of Chemistry and Center for Computational Engineering and Sciences, University of Campinas, 13084-862, SP, Brazil

[c] Department of Chemistry and Biochemistry and BioFrontiers Institute, University of Colorado, Boulder CO 80303 USA

[d] Biosciences Center, National Renewable Energy Laboratory, Golden CO 80403 United States

* To whom correspondence should be addressed: gregg.beckham@nrel.gov, zhongping.tan@colorado.edu, michael.crowley@nrel.gov

Table of Contents

1. Supplementary Methods

1.1. Experimental material and equipment.

1.2. General procedure for the synthesis of linker variants.

1.3. General procedure for the thermolysin digestion of linker variants.

1.4. Temperature replica exchange molecular dynamics simulations.

2. Supplementary Tables

Table S1. Persistence length of protein backbone.

Table S2. Surface of contact between glycans and protein.

Table S3. Root-mean-square deviation analysis and number of clusters from simulations of *Tr*Cel7A linkers.

3. Supplementary Figures

Figure S1. LC-MS traces and ESI-MS of L_{NG}.

Figure S2. LC-MS traces and ESI-MS of L_{man}.

Figure S3. LC-MS traces and ESI-MS of L_{gal}.

Figure S4. LC-MS traces and ESI-MS of L_{glc}.

Figure S5. Predicted sites of cleavage.

Figure S6. Solvent accessible surface area of the variants of *Tr*Cel7A linker.

Figure S7. Clustering analysis of linker structures from simulations.

Figure S8. Ramachandran plots of each threonine residue of variants of *Tr*Cel7A linker.

Figure S9. Simulated models.

Figure S10. Evolution in the free energy profile relative to end-to-end distance for $L_{man\text{-}h.}$

Figure S11. Free energy profiles as a function of end-to-end distance for variants with oligosaccharides.

Figure S12. Hydrogen bond interactions and glycan orientation in variants of PT linker.

Figure S13. Hydrogen bond interactions and glycan orientation in tripeptides.

4. Supplementary References

1. Supplementary methods

1.1. Experimental material and equipment

All commercial reagents and solvents were used as received. All LC-MS analyses were performed using a Waters Acquity™ Ultra Performance LC system equipped with Acquity UPLC® BEH 300 C4, 1.7µm, 2.1 x 100 mm column at flow rates of 0.3 mL/min. The mobile phase for LC-MS analysis was a mixture of H_2O (0.1% formic acid, v/v, A) and acetonitrile (0.1% formic acid, v/v, B). All preparative separations were performed using a LabAlliance HPLC solvent delivery system equipped with a Rainin UV-1 detector and a Vydac C18 250x10mm column (218TP1010) at a flow rate of 5.0 mL/min. The mobile phase for HPLC purification was a mixture of H_2O (0.05% TFA, v/v, A) and acetonitrile (0.04% TFA, v/v, B). Mass spectrometric analysis done with a Waters SYNAPT G2-S LC-MS system was used to confirm the identity and homogeneity of the synthetic peptides. MALDI-TOF mass spectrometric analysis done with an AB/Sciex Voyager DE-STR system was used to determine the proteolytic stability (half-life to thermolysin degradation) of the synthetic peptides (Fig. S1-S4). The glycoamino acid building blocks Fmoc-Ser(Ac_4Manα)-OH, Fmoc-Thr(Ac_4Manα)-OH, Fmoc-Ser(Ac_4Galα)-OH, Fmoc-Thr(Ac_4Galα)-OH, Fmoc-Ser(Ac_4Glcα)-OH, and Fmoc-Thr(Ac_4Glcα)-OH were synthesized following previously described procedures.[1]

1.2. General procedure for the synthesis of linker variants

Synthesis of non-glycosylated linker. Automated peptide synthesis was performed on an Applied Biosystems Pioneer continuous flow peptide synthesizer. Peptides were synthesized under standard

automated Fmoc conditions. The deblock solution was a mixture of 100/5/5 (v/v) of DMF/piperidine/DBU. Fmoc protected amino acid (4.0 eq.), HATU (4.0 eq.) and DIEA (8.0 eq.) were used for the coupling steps. The Fmoc-Pro-NovaSyn® TGT resin from EMD Millipore and the amino acids from Chem-Impex International, including Fmoc-Ala-OH, Fmoc-Gly-OH, Fmoc-Asn(Trt)-OH, Fmoc-Pro-OH, Fmoc-Arg(Pbf)-OH, Fmoc-Ser(tBu)-OH, and Fmoc-Thr(tBu)-OH, were used for the synthesis. Upon completion of automated synthesis on a 0.05 mmol scale, the peptide resin was washed into a peptide cleavage vessel with DCM. The resin cleavage was performed by treatment with 10 mL of TFA/TIS/H_2O (95/2.5/2.5, v/v) at room temperature for 45 min. The resin was filtered and washed with 10 mL of TFA/TIS/H_2O (95/2.5/2.5, v/v). The filtrate was combined and the solvent was removed with compressed air. The oily residue was precipitated with diethyl ether and centrifuged to give a white pellet. The ether was decanted. The solid was dissolved in 10 mL of acetonitrile/H_2O (50/50, v/v) and was lyophilized to dryness. 105 mg of crude peptide was obtained in 83% yield. 30 mg of crude product was dissolved in 4 mL of H_2O/acetonitrile (95:5, v/v) for HPLC purification. After HPLC purification with a linear gradient of 5-25% B over 30 min (Vydac C18 column, 230 nm wavelength) and lyophilization, 8.1 mg pure product was obtained in 27% yield.

Synthesis of glycosylated linkers. The solid-phase synthesis of each glycosylated linker (Fig. 1) was conducted similar to the non-glycosylated linker except that Fmoc-Ser(Ac_4Manα)-OH, Fmoc-Thr(Ac_4Manα)-OH, Fmoc-Ser(Ac_4Galα)-OH, Fmoc-Thr(Ac_4Galα)-OH, Fmoc-Ser(Ac_4Glcα)-OH, and Fmoc-Thr(Ac_4Glcα)-OH were used to introduce desired sugars to the peptides as building blocks. The crude glycosylated peptides could be obtained in 21-35% yield (62 mg of L_{man} in 23% yield; 59 mg of L_{gal} in 21% yield; 97 mg of L_{glc} in 35% yield). 30 mg of the crude peptide was dissolved in 1.5 mL of hydrazine solution (hydrazine/H_2O, 5/100, v/v) and stirred at room temperature for 30 min under helium. The reaction was quenched with 3 mL of acetic acid solution (AcOH/H_2O, 5/100, v/v). After HPLC purification with a linear gradient of 0-20% B over 30 min (Vydac C18 column, 230 nm wavelength)

and lyophilization, the pure product was obtained in 29-44% yield (9.6 mg of L_{man} in 44% yield; 6.4 mg of L_{gal} in 29% yield; 8.9 mg of L_{glc} in 41% yield).

1.3. General procedure for the thermolysin digestion of linker variants

The thermolysin digestions were performed following previously described procedures.[2] The digestions of glycosylated linkers were carried out at 37 °C in 25 μL of digestion buffer (50 mM Tris·HCl buffer, 0.5 mM $CaCl_2$, pH 8.0) with an initial linker concentration of 2 μg/μL. 25 μL of thermolysin (1 μg/μL in the digestion buffer) was added to linker solution; 1 μL aliquots were taken at specific time intervals (0, 2, 5, 10, 15, 20, 30, 45, 60, 90, 120, 180, 300, and 420 min) and quenched with a mixture containing 20 μL of acetonitrile/H_2O/AcOH (20/20/1, v/v) and 3 μL of standard solution [0.17 μg/μL non-glycosylated linker in acetonitrile/H_2O/AcOH (20/20/1, v/v)]. Each sample was analyzed by quantitative MALDI-TOF MS to calculate the change in concentration of glycosylated linkers with time. The digestion rate was determined by monitoring and fitting data to the first-order exponential decay of the full-length glycosylated linkers over time. Digestion and analysis of non-glycosylated linker was done exactly as described above for glycosylated linkers except that the L_{man} was used as the internal standard. All the digestion processes were repeated for three independent trials.

1.4. Temperature replica exchange simulations

Model systems. This work compares the dynamical and structural behavior of four variants of the Cel7A linker from *Trichoderma reesei* (*Tr*Cel7A). This set includes the non-glycosylated linker and the linker homogeneously decorated with *O*-linked α-mannosyl, α-glucosyl, and α-galactosyl moieties (Fig. S9A). Complementary simulations and analyses were also performed with the linker decorated with longer glycans comprising mannobiosyl and galactobiosyl moieties, as well as with the putative natural decoration proposed by Harrison *et al.*[3] (Fig. S9B). A set of four variants of the "PT linker" was also studied, including the non-glycosylated linker and the variants with the same three glycans (Fig. S9C). Finally, we also performed simulations of tripeptides with a single site of glycosylation, resulting in the

total of 15 systems simulated (Fig. S9D).

Each linker variant has the termini capped (acetylated N-terminus and amidated C-terminus) and was individually hydrated in an octahedral box containing sodium and chloride ions (~0.15 M). The excess of chloride ions guarantees electroneutrality in the systems of *Tr*Cel7A linkers. About 16,000, 13,500 and 2,800 water molecules were added to the systems of variants of *Tr*Cel7A linker, PT linker, and tripeptides, respectively. The simulation boxes were built with GROMACS-4.6.1 software package.[4] The CHARMM36 force field[5,6] was used to describe the peptides, CHARMM carbohydrate force field[7,8] to describe glycans and TIP3P[9] to describe water molecules. CHARMM-formatted topology and parameter files were converted to GROMACS input files using the TopoGromacs plugin[10] integrated within the VMD[11] software.

Simulations details. All the simulations were conducted with GROMACS-4.6.1 software.[4] We used the Patriksson and van der Spoel algorithm[12] to define temperature of replicas between 300 and 400 K. The total of 60 replicas were used for the linkers and 28 for the tripeptides. These schemes yielded uniform acceptance rates of 27-30% with swap attempts every 2 ps.[13] Before swapping, the replicas were equilibrated for 100 ns with temperature and pressure control, starting from different conformations. The simulations were carried out under periodic boundary conditions with a 2 fs timestep. Temperature control was performed using velocity rescaling method with a stochastic term that properly generates canonical or constant pressure-constant temperature ensembles.[14] A coupling constant of 0.1 ps was used for the thermostat. The Parrinello-Rahman barostat[15] with coupling constant of 1.0 ps was used with 1 atm as reference. Electrostatic interactions were calculated via the particle mesh Ewald method[16] and a cutoff radius of 12 Å was applied for short-range interactions. Bonds including hydrogen atoms were constrained by the LINCS algorithm.[17]

Each replica of *Tr*Cel7A linkers, PT linkers, and tripeptides (Fig. S9) was run for 250 ns, 200 ns and 100 ns, respectively. The convergence of the free-energy profile as a function of end-to-end distance for a

long simulation (390 ns) of the system with the highest number of degrees of freedom, $L_{man\text{-}h}$, was used as initial reference to determine the simulation time of the linkers. As shown in Fig. S10, there are no substantial changes in the free energy profile by extending the simulations beyond 200 ns. This convergence in the free energy profile was observed for the other systems, and the final curves are depicted in Fig. 2 and 5B in the main text. The error bars computed from bootstrapping suggest the simulations are reliable for this comparative study.

1.5. Simulation analyses

The first 15 ns of the T-REMD simulations was discarded and the remaining trajectory of the replica at 300 K was used for all the analyses. As described below, most of the analyses of the linkers were conducted for the regions predicted to be susceptible to proteolytic cleavage (Fig. S5), that is, residue 11 (T) to residue 21 (T) in the *Tr*Cel7A linkers, and residues 2 (T) to residue 14 (T) in the PT linkers.

Free energy profiles. The free energy profile relative to the end-to-end distance was computed for the glycosylated region of the linkers. The Cα atoms in the ends were used to define the distances. The potential of mean force (PMF) was computed from the histograms of the simulation data collected in 1 Å bins, applying $F/kT = -\ln[r] + C$, where F = PMF, k = Boltzmann's constant, T = temperature, r = bin value, and C is a constant. Bootstrapping error analysis was conducted with software available from Alan Grossfield.[18] The data sets generated from resampling were tuned by the autocorrelation time of end-to-end distance computed for the replica at 300 K. Since the time correlation functions (TCF) were markedly non-exponential and exhibit fast components responsible for most of its decay at short times, we considered the correlation time when TCF has decayed 90 % of its initial value.

Hydrogen bond interactions. The Hbonds plugin in VMD[11] was used to count the hydrogen bond interactions along the simulations. The geometric criteria adopted are a cutoff of 3.0 Å for donor-acceptor distance and 20° for acceptor-donor-H angle.

Relative orientation of glycosyl rings. We computed the distribution of the angles between the normal to

the plane formed by atoms C1, C3, and C5 in the glycosyl ring and the vector formed by Cα and Cβ in the linked threonine residue. The distribution plots are normalized and averaged over the glycosyl-threonine groups.

Ramachandran plots. Dihedral pairs φ and ψ were collected along the simulations for the glycosylated threonines in the linkers. MATLAB[19] was used to make the plots.

Persistence length. The cosine of the angles between bonds with even index difference was computed along the backbone of the "cleavable" region of the protein. The persistence length was defined as the number of bonds (each bond measuring ~0.15 nm) where the average cosine relative to bond difference decays to 1/e. Errors were estimated by block averaging.

Solvent accessible surface area. The area of the protein residues exposed to the solvent was computed using the double cubic lattice method.[20] A probe radius of 0.56 nm, larger than the typical value adopted for a polar solvent probe (0.14 nm), was defined to represent the accessibility to the bulky catalytic residues from a protease.

Clustering analysis. The conformations of the backbone in the cleavable region of the linkers were grouped via an RMSD-based algorithm.[21] We used the root mean square deviation of atom-pair distances with 0.15 nm cut-off as parameter for clustering.

Backbone mobility (root-mean-square deviation). Root-mean-square deviation (RMSD) values relative to average structures of 10 ns trajectory blocks were computed for backbone atoms in the cleavable region of the linkers. The final reported value is the average RMSD over the blocks.

2. Supplementary Tables

Table S1. Persistence length of the region of glycosylation (residues T11 to T21) of *Tr*Cel7A linkers.

Model	Φ [nm]
L_{NG}	0.67 ± 0.01
L_{man}	0.88 ± 0.07
L_{gal}	0.80 ± 0.03
L_{glc}	0.80 ± 0.02

Table S2. Total interfacial area between glycans and protein (probe radius of 0.21 nm) in glycosylated variants of *Tr*Cel7A linker.

Model	Area [nm^2]
L_{man}	4.12 ± 0.27
L_{gal}	4.05 ± 0.30
L_{glc}	4.04 ± 0.30

Table S3. Root-mean-square deviation (RMSD) values and total number of clusters from the simulations of variants of *Tr*Cel7A linker. (**A**) RMSD of backbone atoms relative to the average structures computed for 10 ns trajectory blocks. (**B**) The total number of clusters obtained from the simulations. Clustering analysis of the cleavable region (T11 to T21) was conducted considering the positions of the Cα atoms, using an RMSD-based algorithm,[21] with a cutoff of 1.5 nm.

Model	RMSD [nm]	Clusters
L_{NG}	0.34 ± 0.01	344
L_{man}	0.28 ± 0.01	255
L_{gal}	0.30 ± 0.01	370
L_{glc}	0.32 ± 0.01	349

3. Supplementary Figures

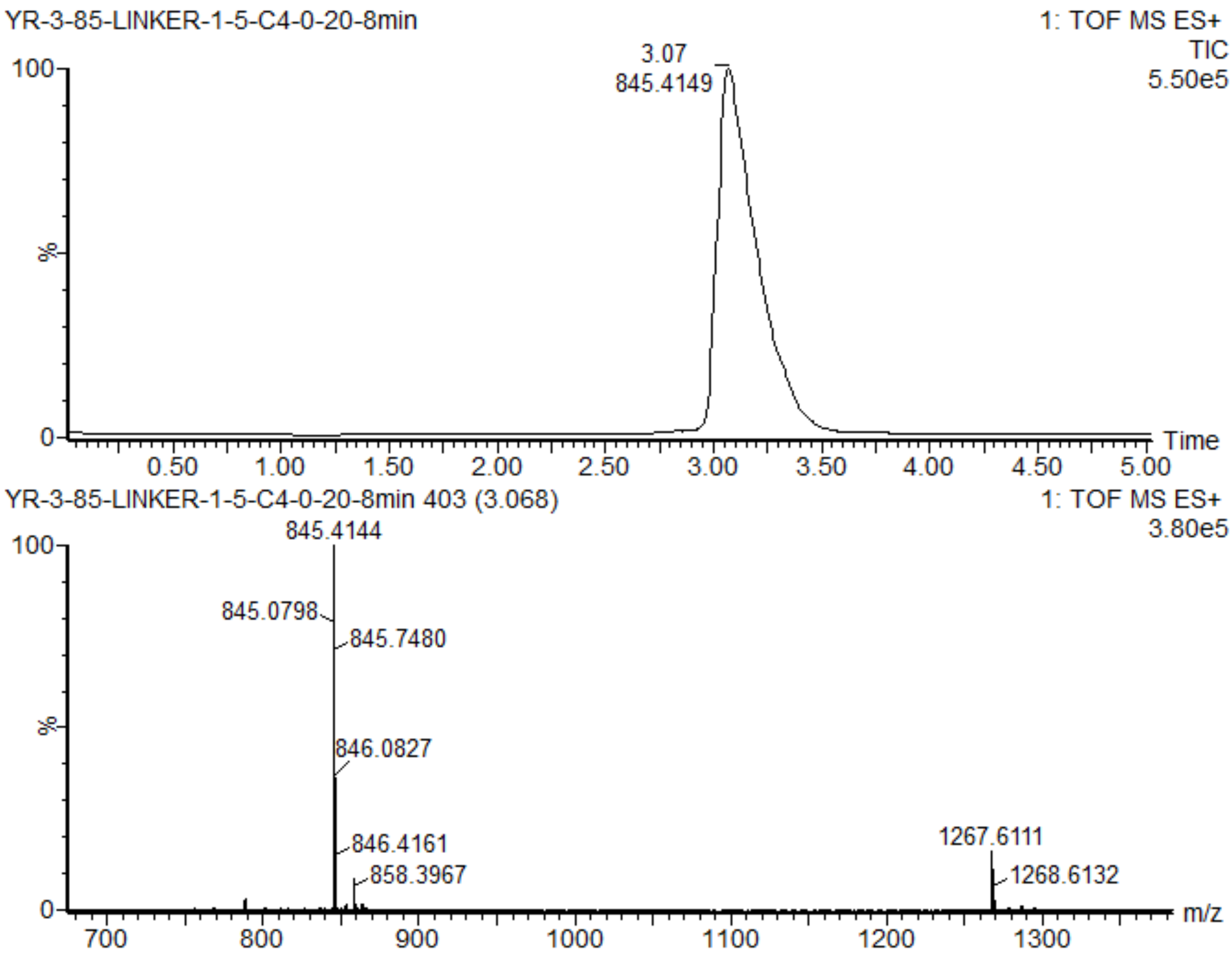

Fig. S1 LC-MS traces and ESI-MS of L_{NG}. MS (ESI) calculated for linker $C_{104}H_{169}N_{35}O_{39}$, exact mass: 2532.2317, $[M+2H]^{2+}$ m/z = 1267.1129 Da, $[M+3H]^{3+}$ m/z = 845.0772 Da.

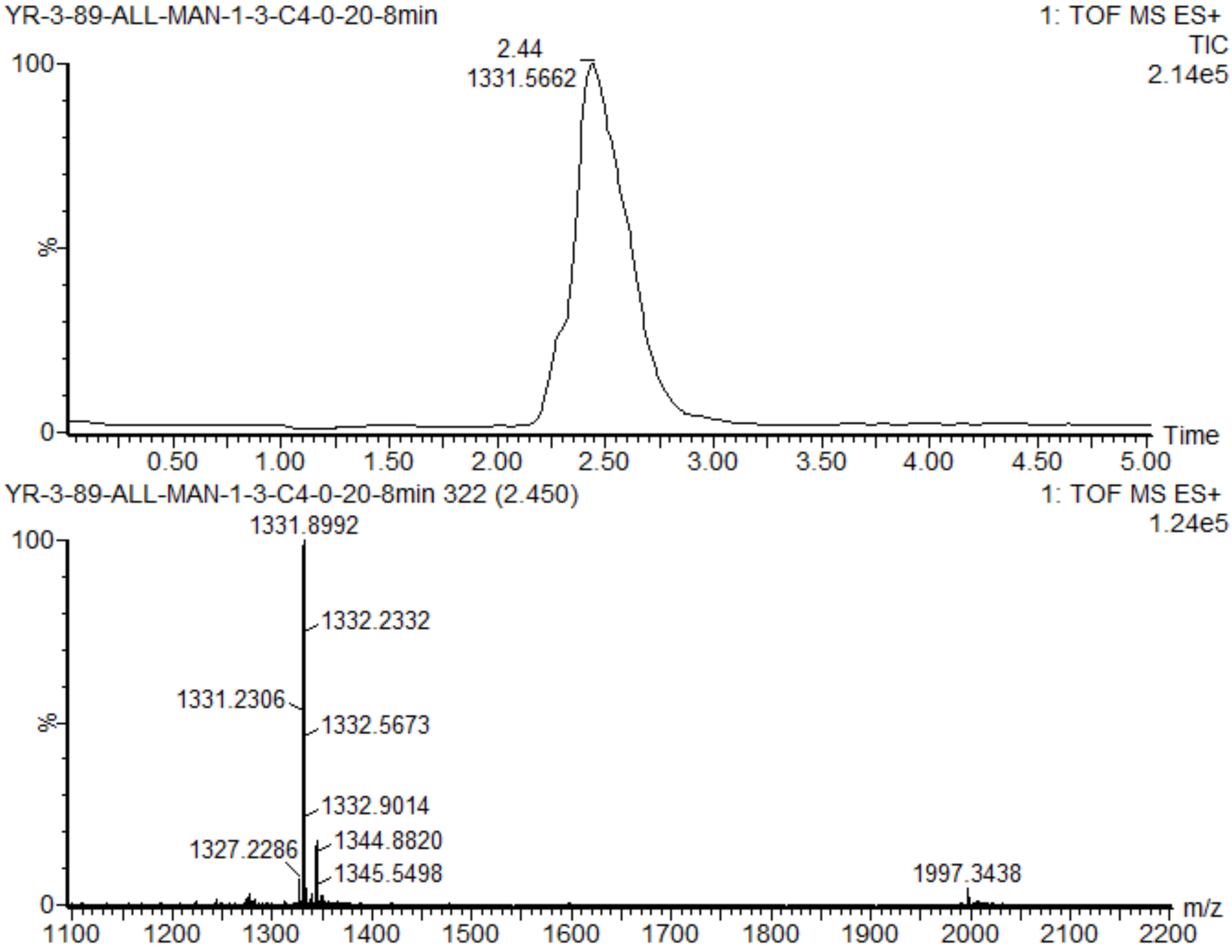

Fig. S2 LC-MS traces and ESI-MS of L_{man}. MS (ESI) calculated for L_{man} $C_{157}H_{258}N_{35}O_{84}$, exact mass: 3977.6993, $[M+2H]^{2+}$ m/z = 1996.3536 Da, $[M+3H]^{3+}$ m/z = 1331.2357 Da.

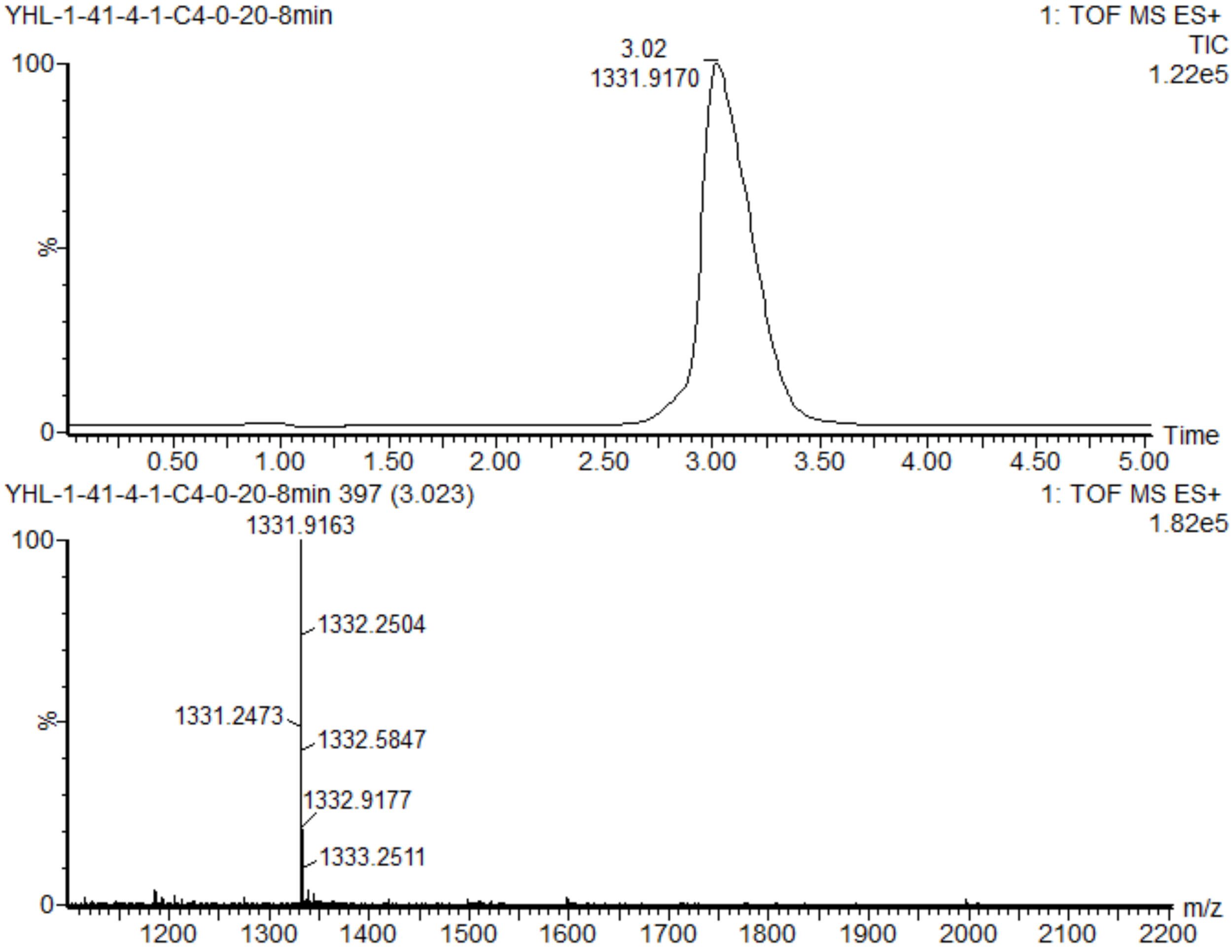

Fig. S3 LC-MS traces and ESI-MS of L_{gal}. MS (ESI) calculated for L_{gal} $C_{157}H_{258}N_{35}O_{84}$, exact mass: 3977.6993, $[M+2H]^{2+}$ m/z = 1996.3536 Da, $[M+3H]^{3+}$ m/z = 1331.2357 Da.

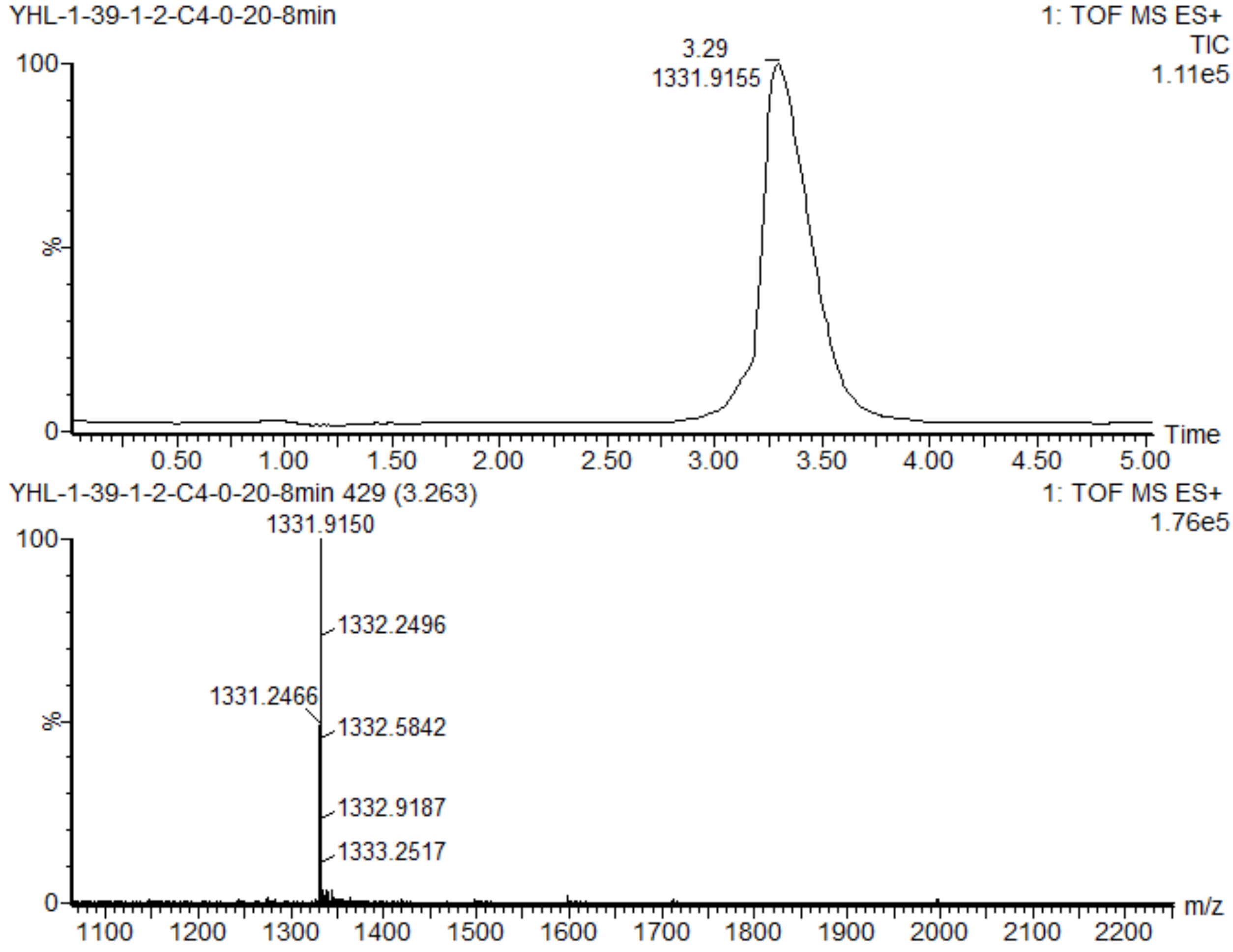

Fig. S4 LC-MS traces and ESI-MS of L_{glc}. MS (ESI) calculated for L_{glc} $C_{157}H_{258}N_{35}O_{84}$, exact mass: 3977.6993, $[M+2H]^{2+}$ m/z = 1996.3536 Da, $[M+3H]^{3+}$ m/z = 1331.2357 Da.

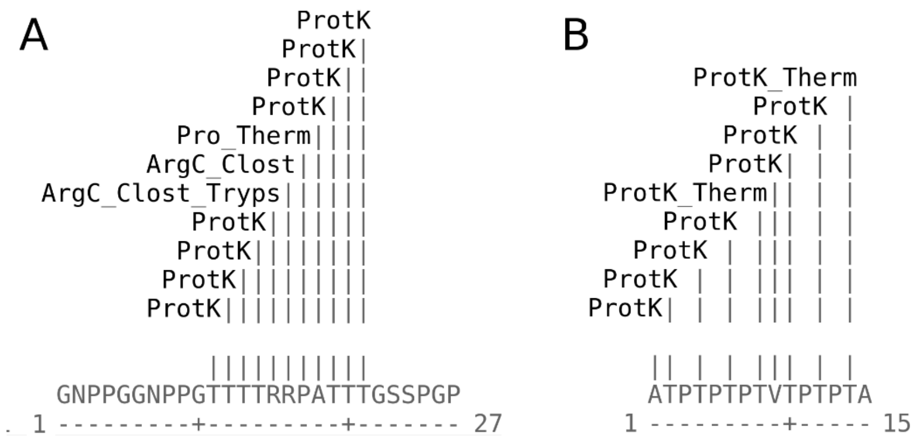

Fig. S5 Sites of proteolytic cleavage predicted by the ExPASy PeptideCutter tool[22] for (**A**) the *T. reesei* Cel7A linker and for (**B**) the PT linker. The proteases, proteinase K (ProtK), proline-endopeptidase (Pro), thermolysin (Therm), Arg-C proteinase (ArgC) and trypsin (Tryps), are assigned in their putative specific site of cleavage.

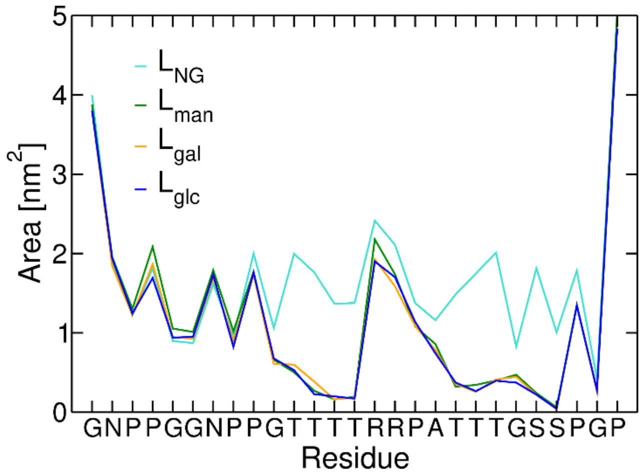

Fig. S6 Solvent accessible surface area of the protein residues in the *T. reesei* Cel7A glycosylated linkers, averaged along the simulations, using probe radius of 0.56 nm.

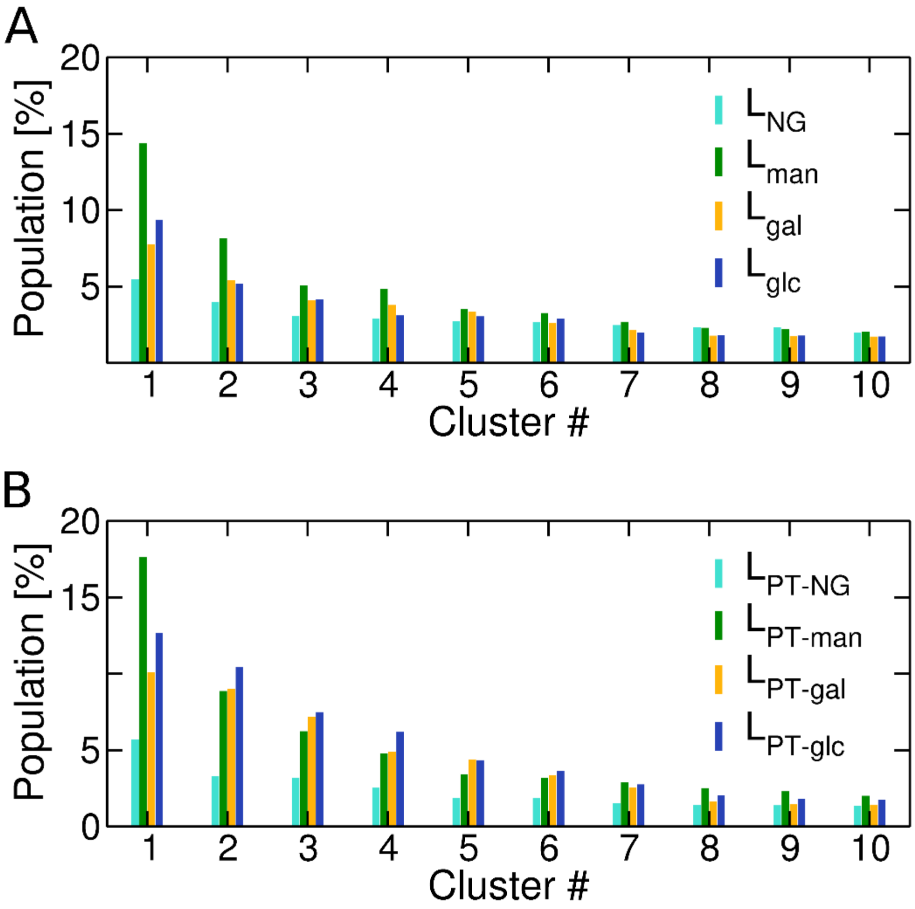

Fig. S7 The ten most populated clusters obtained from the simulations of (**A**) the *Tr*Cel7A linkers and (**B**) the PT linkers. Clustering analysis of the glycosylated region (T11 to T21 in the *Tr*Cel7A linkers and T2 to T14 in the PT linkers) was conducted considering the positions of the Cα atoms, using an RMSD-based algorithm,[21] with a cutoff of 1.5 nm.

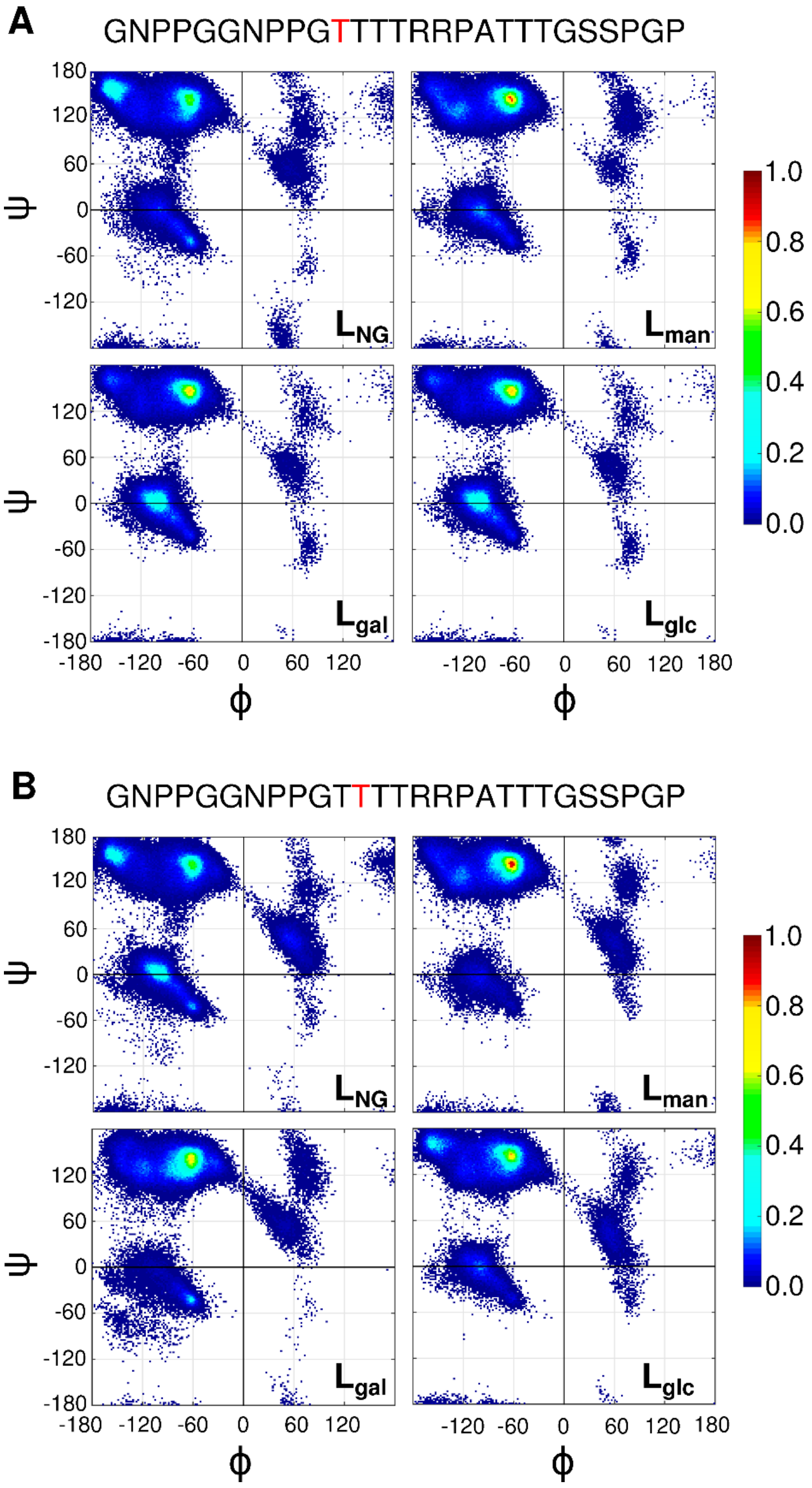
A
GNPPGGNPPGTTTTRRPATTTGSSPGP
ψ
ϕ
L_NG
L_man
L_gal
L_glc
1.0
0.8
0.6
0.4
0.2
0.0
B
GNPPGGNPPGTTTTRRPATTTGSSPGP

C

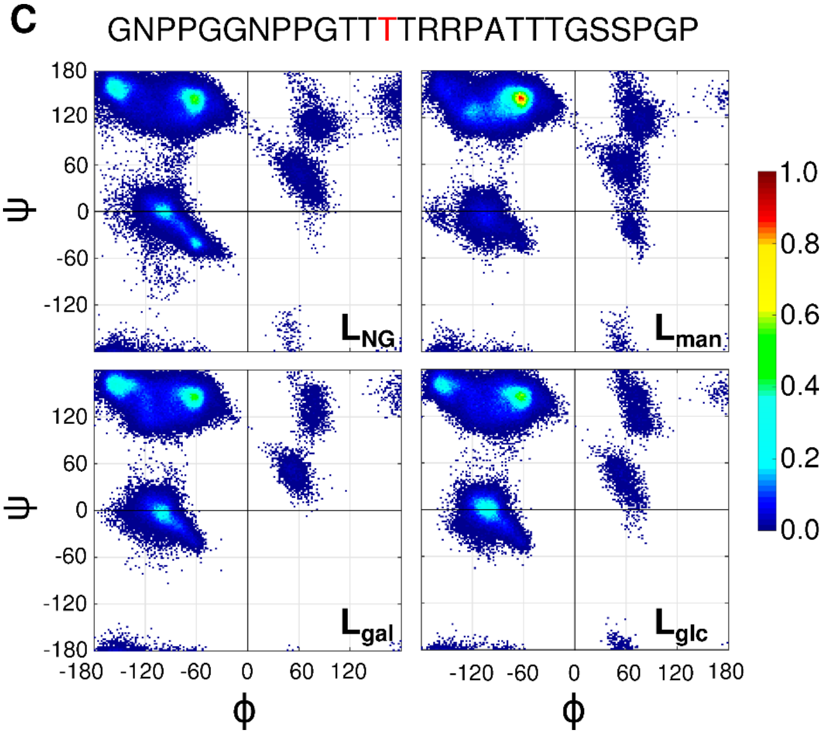
GNPPGGNPPGTTTTRRPATTTGSSPGP
ψ
φ
L_{NG}
L_{man}
L_{gal}
L_{glc}
180
120
60
0
-60
-120
-180
-180 -120 -60 0 60 120 180
1.0
0.8
0.6
0.4
0.2
0.0

D

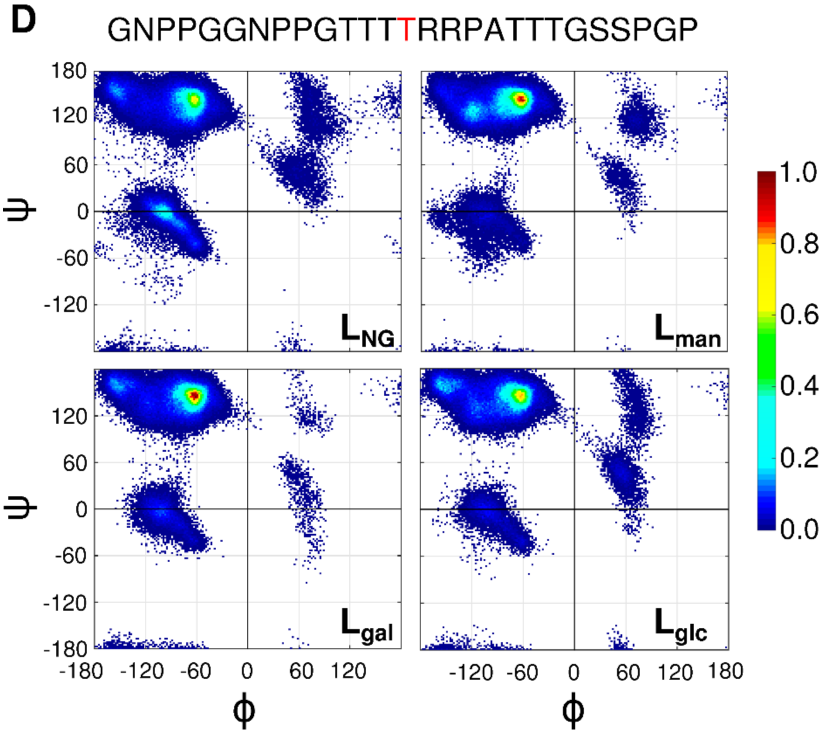
GNPPGGNPPGTTTTRRPATTTGSSPGP
ψ
φ
L_{NG}
L_{man}
L_{gal}
L_{glc}
180
120
60
0
-60
-120
-180
-180 -120 -60 0 60 120 180
1.0
0.8
0.6
0.4
0.2
0.0

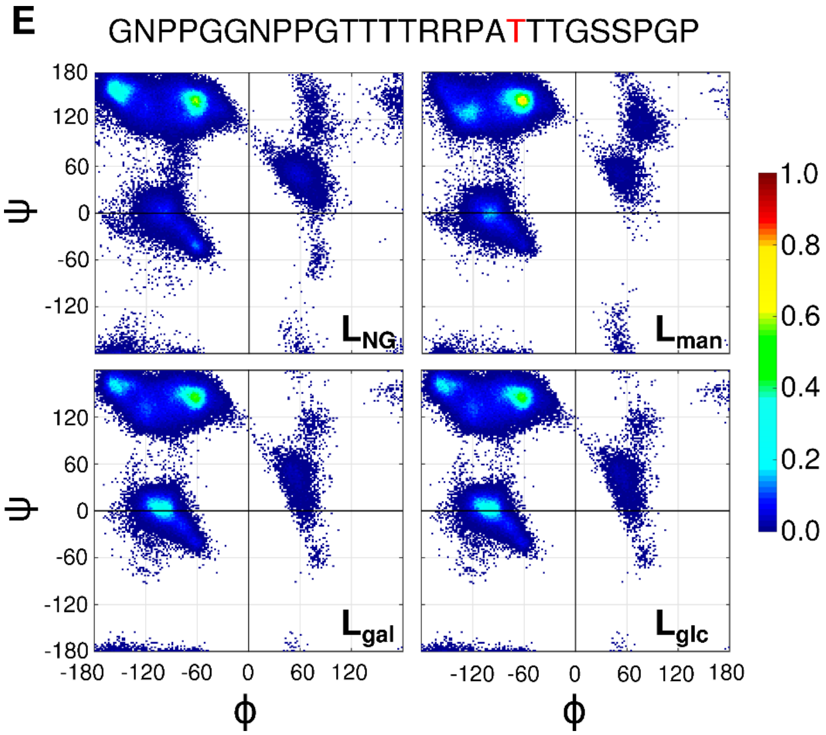
E
GNPPGGNPPGTTTTRRPATTTGSSPGP
180
120
60
0
-60
-120
-180
ψ
L_{NG}
L_{man}
L_{gal}
L_{glc}
-180 -120 -60 0 60 120
-120 -60 0 60 120 180
ϕ
1.0
0.8
0.6
0.4
0.2
0.0

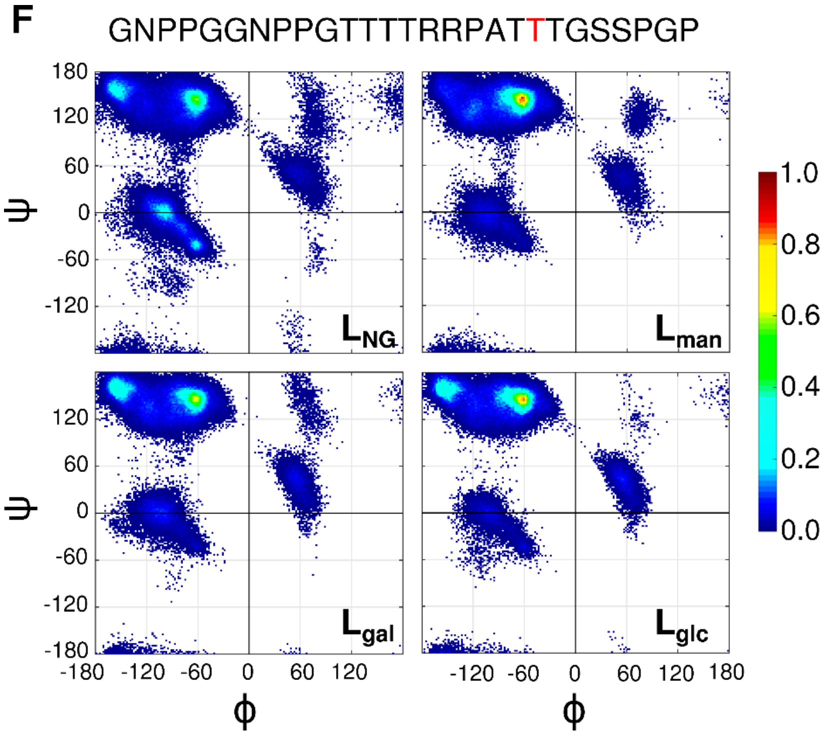
F
GNPPGGNPPGTTTTRRPATTTGSSPGP
180
120
60
0
-60
-120
-180
ψ
L_{NG}
L_{man}
L_{gal}
L_{glc}
-180 -120 -60 0 60 120
-120 -60 0 60 120 180
ϕ
1.0
0.8
0.6
0.4
0.2
0.0

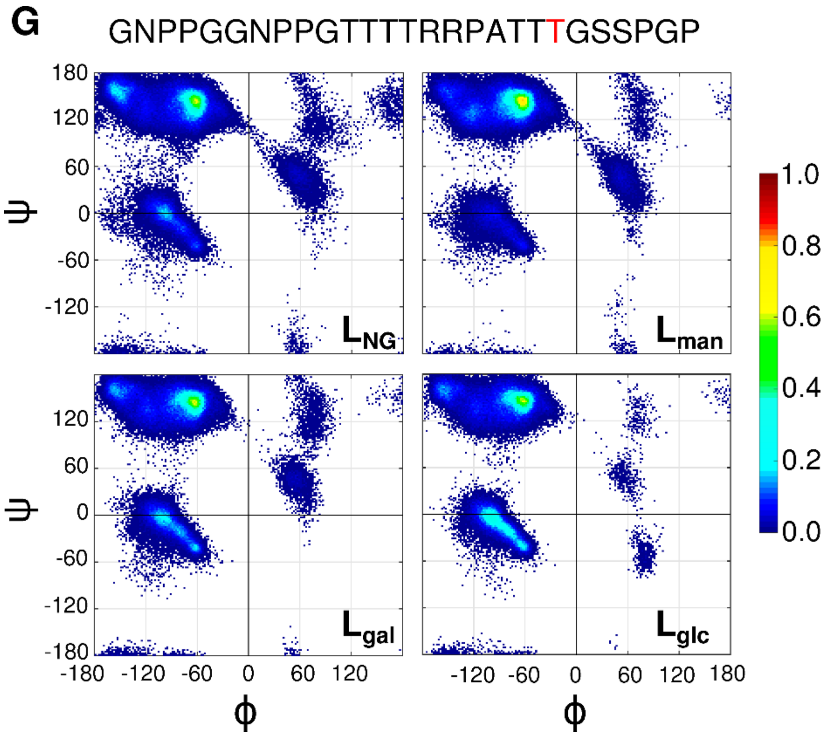

Fig. S8 Ramachandran plots of each threonine (**A-G**) computed from the simulations of variants L_{NG}, L_{man}, L_{gal}, and L_{glc}. All the maps are on the same scale, with the colors representing relative population in the simulations.

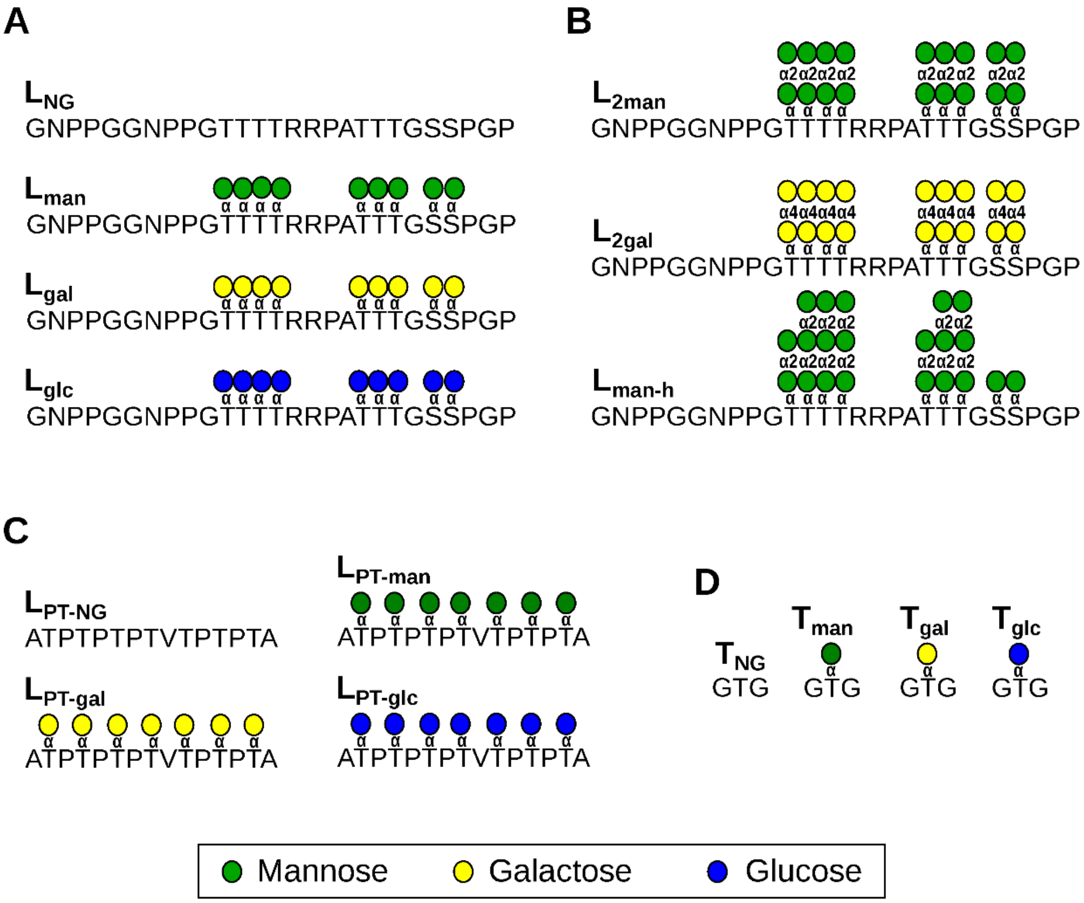

Fig. S9 Non-glycosylated and glycosylated peptides simulated in this work. (**A**) Non-glycosylated *T. reesei* Cel7A linker (L_{NG}) and variants glycosylated with monosaccharide moieties (L_{man}, L_{gal} and L_{glc}). (**B**) *T. reesei* Cel7A linker decorated with longer glycans (L_{2man}, L_{2gal} and $L_{man\text{-}h}$). (**C**) Non-glycosylated ($L_{PT\text{-}NG}$) and glycosylated variants of "PT linker" ($L_{PT\text{-}man}$, $L_{PT\text{-}gal}$ and $L_{PT\text{-}glc}$). (**D**) Short non-glycosylated (T_{NG}) and glycosylated peptides (T_{man}, T_{gal} and T_{glc}). The designated names are written in bold next to the corresponding molecule.

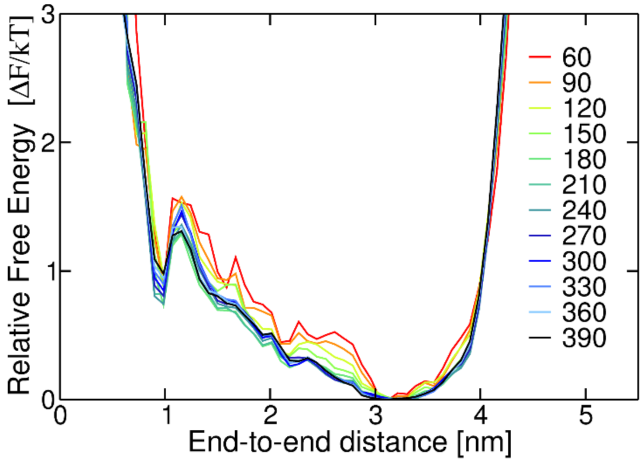

Fig. S10 Evolution in the free energy profile relative to end-to-end distance for L_{man-h} (colors refer to simulation time in nanoseconds).

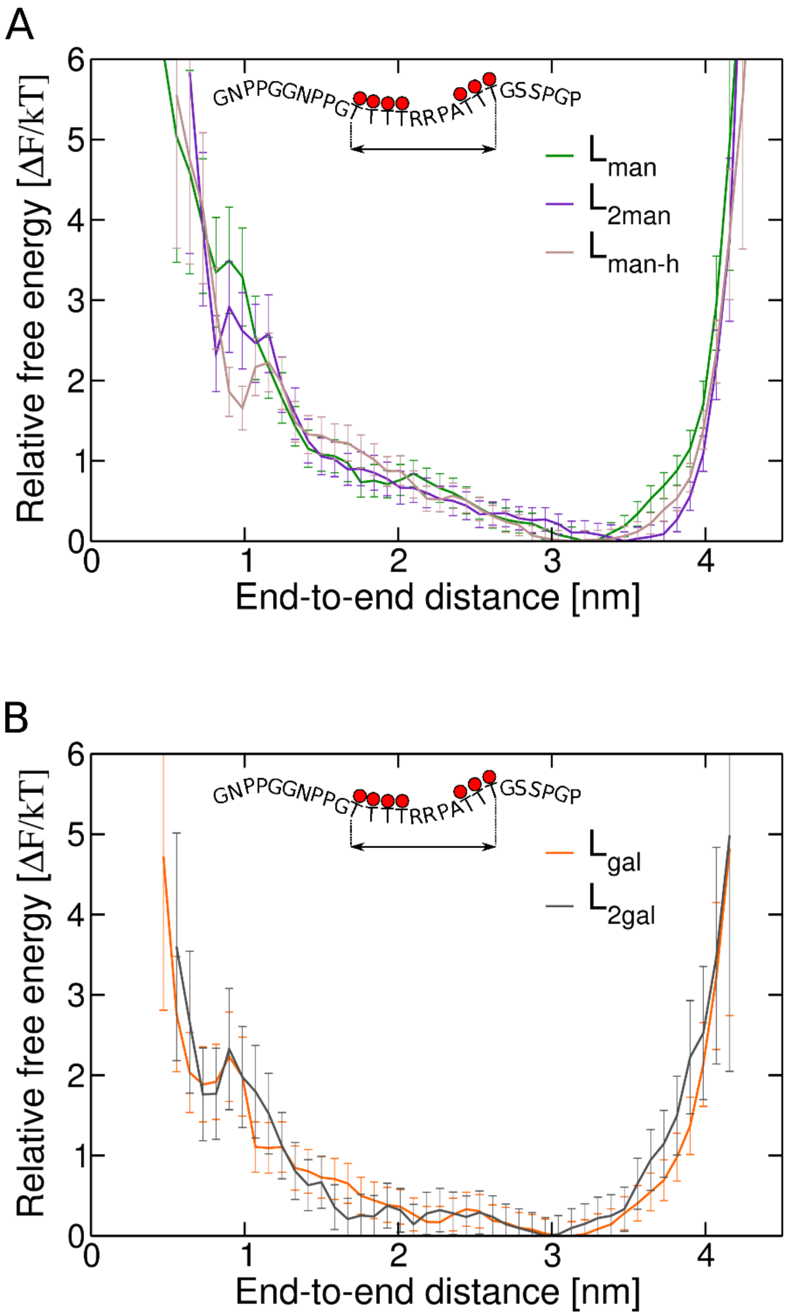

Fig. S11 Free energy profiles as a function of the end-to-end distance of the *Tr*Cel7A linkers decorated with *O*-linked monosaccharides or longer glycans of (**A**) α-mannose and (**B**) α-galactose. Error bars were computed with bootstrapping analysis.

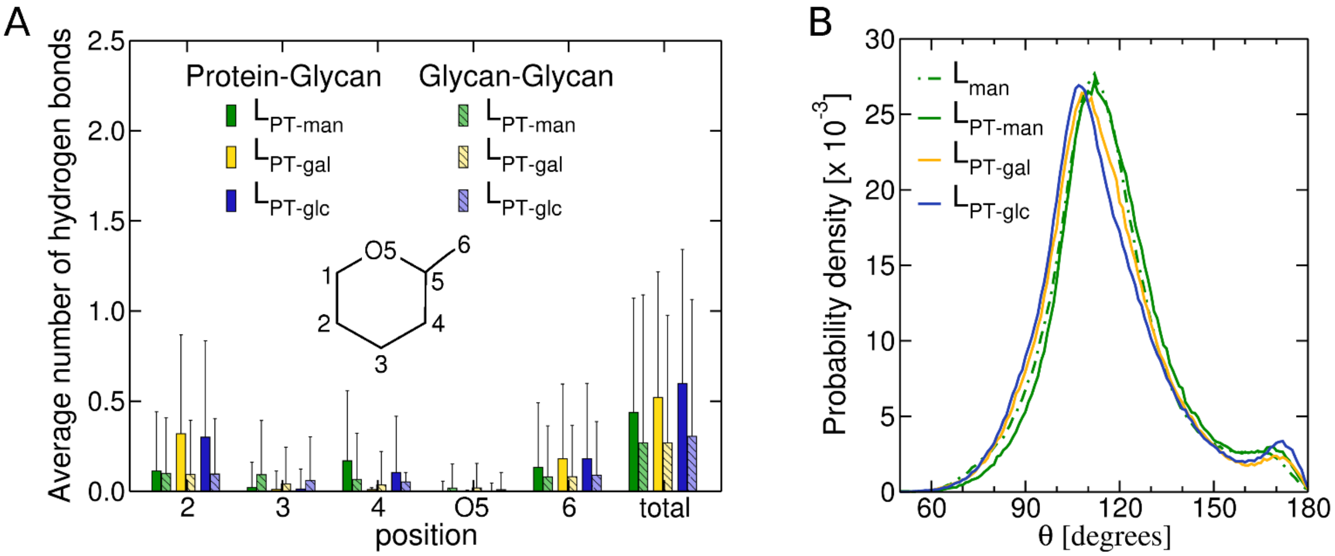

Fig. S12 (**A**) Average number of hydrogen bonds involving hydroxyl groups in the different positions of the carbohydrate ring for PT linkers. The bars with the full colors correspond to interactions with the peptide and the bars with stripes correspond to interactions involving only glycans. Standard deviations are shown with vertical lines; (**B**) Probability distribution of the angle formed between the normal to the plane of the carbohydrate ring and the vector between Cα and Cβ in the threonine to which the glycan is bound.

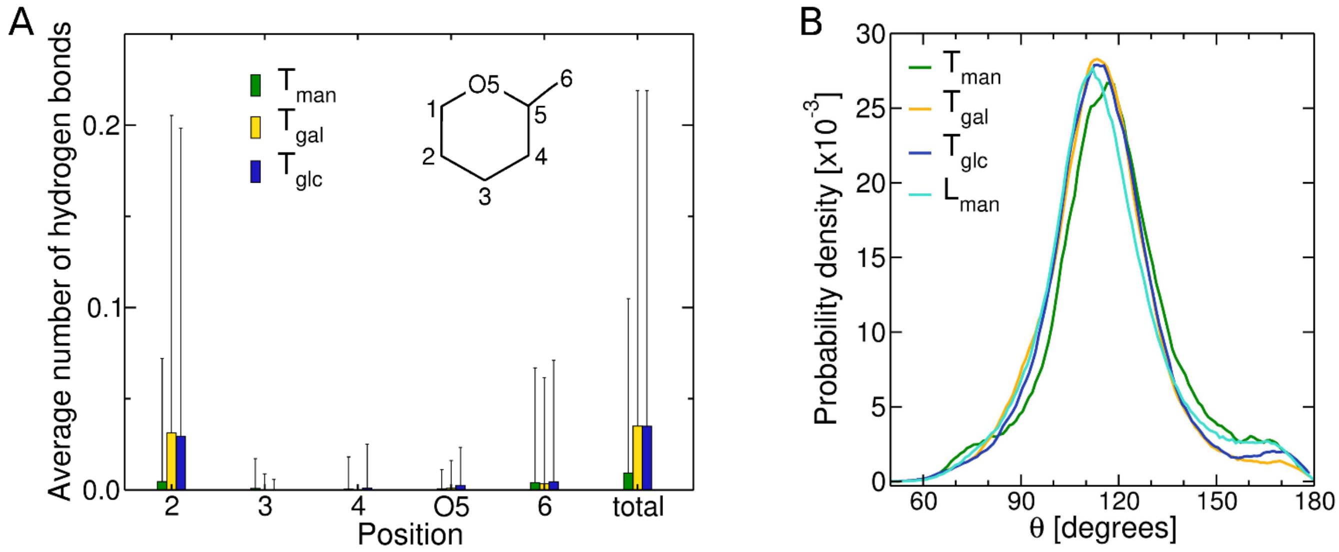

Fig. S13 (**A**) Average number of hydrogen bonds involving hydroxyl groups in the different positions of the carbohydrate ring in the tripeptides. Standard deviations are shown with vertical lines; (**B**) Probability distribution of the angle formed between the normal to the plane of the carbohydrate ring and the vector between Cα and Cβ in the threonine to which the glycan is bound.

4. Supplementary References

1 X. Guan, P. K. Chaffey, C. Zeng, E. R. Greene, L. Chen, M. R. Drake, C. Chen, A. Groobman, M. G. Resch, M. E. Himmel, G. T. Beckham and Z. Tan, *Chem. Sci.*, 2015, **6**, 7185.

2 L. Chen, M. R. Drake, M. G. Resch, E. R. Greene, M. E. Himmel, P. K. Chaffey, G. T. Beckham and Z. Tan, *Proc. Natl. Acad. Sci.*, 2014, **111**, 7612.

3 M. J. Harrison, A. S. Nouwens, D. R. Jardine, N. E. Zachara, A. A. Gooley, H. Nevalainen and N. H. Packer, *Eur. J. Biochem.*, 1998, **256**, 119.

4 B. Hess, C. Kutzner, D. Van Der Spoel and E. Lindahl, *J. Chem. Theory Comput.*, 2008, **4**, 435.

5 R. B. Best, X. Zhu, J. Shim, P. E. M. Lopes, J. Mittal, M. Feig and A. D. MacKerell, *J. Chem. Theory Comput.*, 2012, **8**, 3257.

6 A. D. MacKerell, M. Feig and C. L. Brooks, *J. Am. Chem. Soc.*, 2004, **126**, 698.

7 O. Guvench, S. S. Mallajosyula, E. P. Raman, E. Hatcher, K. Vanommeslaeghe, T. J. Foster, F. W. Jamison and A. D. MacKerell, *J. Chem. Theory Comput.*, 2011, **7**, 3162.

8 O. Guvench, E. R. Hatcher, R. M. Venable, R. W. Pastor and A. D. Mackerell, *J. Chem. Theory Comput.*, 2009, **5**, 2353.

9 W. L. Jorgensen, J. Chandrasekhar, J. D. Madura, R. W. Impey and M. L. Klein, *J. Chem. Phys.*, 1983, **79**, 926.

10 J. V. Vermaas, D. J. Hardy, J. E. Stone, E. Tajkhorshid and A. Kohlmeyer, *J. Chem. Inf. Model.*, 2016, **56**, 1112.

11 W. Humphrey, A. Dalke and K. Schulten, *J. Mol. Graph.*, 1996, **14**, 33.

12 A. Patriksson and D. Van Der Spoel, *Phys. Chem. Chem. Phys.*, 2008, **10**, 2073.

13 D. J. Sindhikara, J. E. Emerson and A. E. Roitberg, *J. Chem. Theory Comput.*, 2010, **6**, 2804.

14 G. Bussi, D. Donadio and M. Parrinello, *Phys. Chem. Chem. Phys.*, 2011, **13**, 13709.

15 M. Parrinello and A. Rahman, *Jounal Appl. Phys.*, 1981, **52**, 7182.

16 T. Darden, D. York and L. Pedersen, *J. Chem. Phys.*, 1993, **98**, 10089.

17 B. Hess, H. Bekker, H. J. C. Berendsen and J. G. E. M. Fraaije, *J. Comput. Chem.*, 1997, **18**, 1463.

18 A. Grossfield, WHAM: the weighted histogram analysis method, version 2.0.9, http://membrane.urmc.rochester.edu/content/wham, (accessed 1 January 2017).

19 MATLAB R2016a, The MathWorks Inc., Natick MA, 2016.

20 F. Eisenhaber, P. Lijnzaad, P. Argos, C. Sander and M. Scharf, *J. Comput. Chem.*, 1995, **16**, 273.

21 X. Daura, K. Gademann, B. Jaun, D. Seebach, W. F. Van Gunsteren and A. E. Mark, *Angew. Chem. Int. Ed*, 1999, **38**, 236.

22 E. Gasteiger, C. Hoogland, A. Gattiker, S. Duvaud, M. R. Wilkins, R. D. Appel and A. Bairoch, in *The proteomics protocols handbook*, ed. J. M. Walker, Totowa, NJ, 2005.
